# The MAPK Signaling Cascade is a Central Hub in the Regulation of Cell Cycle, Apoptosis and Cytoskeleton Remodeling by Tripeptidyl-Peptidase II

**DOI:** 10.4137/grsb.s882

**Published:** 2008-11-24

**Authors:** Ramakrishna Sompallae, Vaia Stavropoulou, Mathieu Houde, Maria G. Masucci

**Affiliations:** 1 Department of Cell and Molecular Biology, Karolinska Institutet, Stockholm, Sweden

**Keywords:** TPPII, MAPK signaling, centrosome, cell cycle, cytoskeleton

## Abstract

Tripeptidyl-peptidase II (TPPII) is a serine peptidase highly expressed in malignant Burkitt’s lymphoma cells (BL). We have previously shown that overexpression of TPPII correlates with chromosomal instability, centrosomal and mitotic spindle abnormalities and resistance to apoptosis induced by spindle poisons. Furthermore, TPPII knockdown by RNAi was associated with endoreplication and the accumulation of polynucleated cells that failed to complete cell division, indicating a role of TPPII in the cell cycle. Here we have applied a global approach of gene expression analysis to gain insights on the mechanism by which TPPII regulates this phenotype. mRNA profiling of control and TPPII knockdown BL cells identified one hundred and eighty five differentially expressed genes. Functional categorization of these genes highlighted major physiological functions such as apoptosis, cell cycle progression, cytoskeleton remodeling, proteolysis, and signal transduction. Pathways and protein interactome analysis revealed a significant enrichment in components of MAP kinases signaling. These findings suggest that TPPII influences a wide network of signaling pathways that are regulated by MAPKs and exerts thereby a pleiotropic effect on biological processes associated with cell survival, proliferation and genomic instability.

## Introduction

TPPII is a 138-kDa serine peptidase found in both cytoplasmic and membrane associated oligomeric complexes of more than 1000 kDa (Balow et al. 1983; Balow et al. 1986; Geier et al. 1999; Rose et al. 1996). The assembled enzyme functions as an exopeptidase that removes tripeptides from the free N-terminus of polypeptides (Balow and Eriksson, 1987; Balow et al. 1983; Balow et al. 1986; Tomkinson et al. 1997), but also exhibits endopeptidase activity towards long peptides (Geier et al. 1999; Seifert et al. 2003). We have previously demonstrated that overexpression of TPPII in Burkitt’s lymphoma or transfected HEK293 cells correlates with accelerated proliferation and with the accumulation of centrosome and chromosome aberrations, whereas functional knockdown of TPPII by shRNA results in growth retardation and the accumulation of polynucleated cells that fail to complete cell division (Stavropoulou et al. 2005). We have also shown that TPPII overexpressing cells evade mitotic arrest induced by spindle poisons and display high levels of polyploidy despite the constitutively high expression of major components of the spindle checkpoints (Stavropoulou et al. 2006). This was accompanied by up-regulation of inhibitors of apoptosis (IAPs) and resistance to p53-induced apoptosis, suggesting that TPPII may allow the transit through mitosis and the survival of cells with severe mitotic spindle damage.

Collectively, these findings suggest that TPPII participates in the regulation of critical events that control the homeostasis of cell division. In particular, the accumulation of centrosome abnormalities and multipolar spindles, together with the capacity to overcome spindle checkpoints, point to a possible involvement of TPPII in the early phases of mitosis, at or around the time of centrosome duplication. A growing body of evidence indicates that, apart from its function as microtubule organization center (MTOC), the centrosome also serves as a scaffold for multiple signaling networks that control critical cellular functions, including the cell cycle, the activation of mitotic checkpoints and stress responses, through association with key regulators such as kinases and motor proteins (Doxsey et al. 2005; Hong et al. 2007).

In the present study, we have performed a global gene expression profiling in cells where TPPII expression was silenced by shRNA to identify regulatory pathways and cellular functions that are affected by modulation of TPPII expression. Computational strategies were then used to correlate transcriptional changes with protein function and physical interaction data in order to interpret the biological activity of the affected cellular pathways. Using this approach we have identified significant changes in the expression levels of genes whose products control fundamental cellular processes including the cell cycle, apoptosis, signal transduction and cytoskeleton remodeling. Amongst the affected genes were several components of the MAPK signaling pathway.

## Materials and Methods

### Cell lines

The Epstein-Barr virus (EBV)-positive Burkitt’s lymphoma (BL) line Namalwa was established from an EBV positive BL biopsy (Klein et al. 1972). The cells were maintained in RPMI medium supplemented with 10% (v/v) fetal calf serum (FCS) at 37 °C in a 5% (v/v) CO_2_ incubator.

### Lentiviruses and infection procedure

Recombinant lentiviruses expressing control and human TPPII gene specific shRNAs were described previously (Stavropoulou et al. 2005). Namalwa cells were plated at a density of 0.5 × 10^6^ cells/well in 6-well plates and were infected with 500 μl of virus stock for 2 h at 37 °C in a 5% CO_2_ incubator and then selected in medium containing 5 μg/ml puromycin (Sigma-Aldrich, Missouri, U.S.A). TPPII protein levels and activity were monitored by western blotting and enzymatic assays as described earlier (Stavropoulou et al. 2005). More than 90% knockdown was usually observed within 10 days of selection.

### RNA isolation

RNA isolation was done according to the Qiagene RNAeasy protocol (Qiagene, Valencia, CA, U.S.A). Briefly, the cells were washed with Phosphate-buffered saline (PBS) and lysed with Qiagene solution containing β-mercaptoethanol. After purification on a Qiagene column, the RNA was eluted and analyzed for quantification and purity with Agilent 2100 bioanalyzer.

### Microarray analysis

High-density oligonucleotide microarrays from Affymetrix were used in this study. cDNA was synthesized using a T7-linked oligo-dT primer and used for cRNA synthesis with biotinylated-UTP and CTP. After fragmenting, the labeled RNAs were hybridized to HG-U133 Plus 2.0 oligonucleotide arrays (Affymetrix Incorporated, Santa Clara, CA, U.S.A.) according to the protocol recommended by the supplier. The arrays were then washed and developed with streptavidin-phycoerythrin and biotinylated antibody against streptavidin (Molecular Probes Inc., Eugene, OR, U.S.A.) in an Affymetrix fluidics station. The HG-U133 Plus 2.0 arrays monitors the expression levels of 47000 transcripts of human genes involved in a wide spectrum of cellular functions.

Hybridized and developed arrays were scanned and the expression values for each probe set were calculated using the Affymetrix Microarray Suite Software (MAS) version 5.0. Scan quality was assured based on *a priori* quality control criteria, including the absence of visible microarray artifacts, significant differences in microarray intensity, and a minimum of 1000 probe sets receiving ‘Present’ calls. Probe level quality control parameters were assessed as indicated in the Affymetrix toolbox and the expression data from three replicates were normalized for further analysis.

Normalized probe set data from each pair of TPPII knockdown and control cells were compared to find differentially regulated genes. The magnitude and direction of expression changes were estimated as Signal Log Ratio (SLR), where an SLR of 1.0 corresponds to a 2-fold increase of the transcript level while SLR −1.0 corresponds to a 2-fold-decrease. Differentially regulated genes were classified into molecular functions according to their Affymetrix annotations or assigned Gene Ontology (GO) categories (http://www.geneontology.org/). WEB-based GEne SeT AnaLysis Toolkit (WebGestalt) (Zhang et al. 2005) was used to identify GO categories and Kyoto Encyclopedia of Genes and Genomes (KEGG) pathways that are significantly enriched of differentially regulated genes. Protein-protein interaction data from Biological General Repository for Interaction Datasets (BioGRID) version 2.0.31(Stark et al. 2006) were used to infer molecular networks involving genes with altered expression. Cytoscope version 2.5.1 was used to visualize the interaction network (Shannon et al. 2003).

## Results

### Functional knockdown of TPPII by shRNA

**Figure 1:**
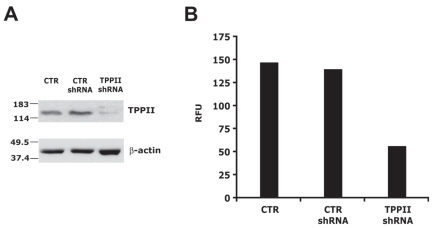
TPPII expression and activity is efficiently suppressed by TPPII specific shRNA **A**) Representative Western blot illustrating the expression of TPPII in untreated Namalwa cells (CTR) and Namalwa cells transduced with lentivirus expressing control (CTR shRNA) or TPPII specific shRNAs (TPPII shRNA) after 10 days on puromycin selection. Chicken antibodies specific for TPPII were used for the detection. **B**) Peptidase activity was assessed by cleavage of the fluorogenic substrates Ala-Ala-Phe-AMC. Activity is expressed as Relative Fluorescence Units (RFU) of free AMC released by incubation for 1 hr at 37 °C in the presence 1 μg of cell lysate.

In order to gain insights on the cellular functions that are regulated by TPPII, global gene expression analysis was performed in the BL line Namalwa, that spontaneously expresses high levels of TPPII, and in Namalwa cells where TPPII expression was silenced by shRNA. Three independent pairs of mRNAs were isolated from cells transduced with previously characterized control and TPPII-specific shRNA expressing lentiviruses. In each case, the efficiency of TPPII knockdown was confirmed before RNA isolation by Western blot analysis using a TPPII specific antibody (Fig. 1A) and by enzymatic assays of total cell lysates using the fluorogenic substrate AAF-AMC (Fig. 1B). The knockdown was confirmed by analysis of the microarrays where the TPPII coding sequence was detected with two Probe sets (203374_s_at and 203375_s_at). TPPII mRNA expression levels scored an average of −1.5 SLR (Signal Log Ratio) corresponding to 2.8 fold down-regulation.

### Microarray data analysis and gene filtering

**Figure 2:**
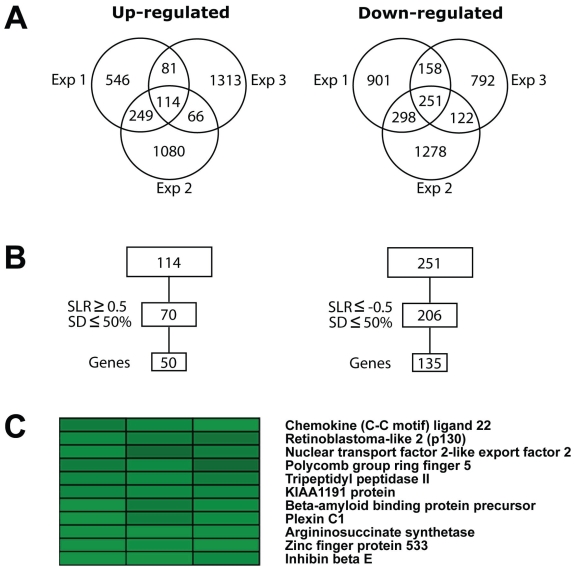
Statistical analysis of gene expression data **A**) Venn diagram demonstrating the number of probe sets “Up-regulated” and “Down-regulated” by TPPII knockdown in three independent experiments. **B**) The probe sets were filtered with cut-off signal log ratios (SLR) of ≥0.5 (for up-regulated) or ≤ −0.5 (for down-regulated) and standard deviation (SD) less than 50% between three experiments and a single representative probe set was then used. **C**) TPPII was found in the cluster of highly down-regulated genes with an average of 2.8 fold repression.

Gene expression profiles were obtained using Affymetrix GeneChips HG-U133 Plus 2.0 arrays. Microarray data were subjected to quality test and then normalized for further analysis. Pair-wise comparisons between control and TPPII knockdown samples were performed between each Probe set on one array and its counterpart on the second array, and a Difference Call was obtained, indicating “Increase”, “Decrease” or “No Change”. One hundred and fourteen probe sets showing “Increase” in all three experiments and 251 “Decrease” (Fig. 2A) probes were further filtered based on reproducibility between experiments. Probe sets showing SLR ≥0.5 or ≤ −0.5 in each experiment and standard deviation ≤50% between the three experiments were identified as stringently modulated. Multiple probe sets for the same gene were than reduced to one representative probe set. One hundred and eighty five genes displayed significant transcriptional changes upon TPPII knockdown, 50 were up-regulated and 135 down-regulated (Fig. 2B). Cluster analysis was performed to identify the genes with similar expression patterns. As expected, TPPII was found in the cluster of the most significantly down-regulated genes (Fig. 2C). Inhibin beta E (INHBE), Plexin C1, Retinoblastoma-like 2 (p130) and a few hypothetical proteins were also present in this cluster. The most down-regulated gene was INHBE, showing a 6.3 fold decrease in TPPII knockdown cells (Fig. 2C). Similar levels of downregulation were detected by RT-PCR analysis confirming the validity of the microarray data (not shown). INHBE is a secreted protein belonging to the Tumor growth factor-β (TGF-β) family and was earlier shown to be involved in the regulation of liver homeostasis and proliferation of pancreatic exocrine cells (Chabicovsky et al. 2003; Hashimoto et al. 2006). The cluster of highly up-regulated genes included the cytoskeleton regulators Actin like protein 8 (ACTL8) and Myosin binding protein C1 (MYBPC1) up-regulated 5.9 and 3.8 fold respectively. Macrophage expressed gene 1 (MPEG1), a Mps1 protein family member that participates in centrosome duplication and spindle assembly (Fisk et al. 2003), was up-regulated 3.6 fold (Supplementary information Table S1).

### Functional classification and gene ontology analysis

**Figure 3:**
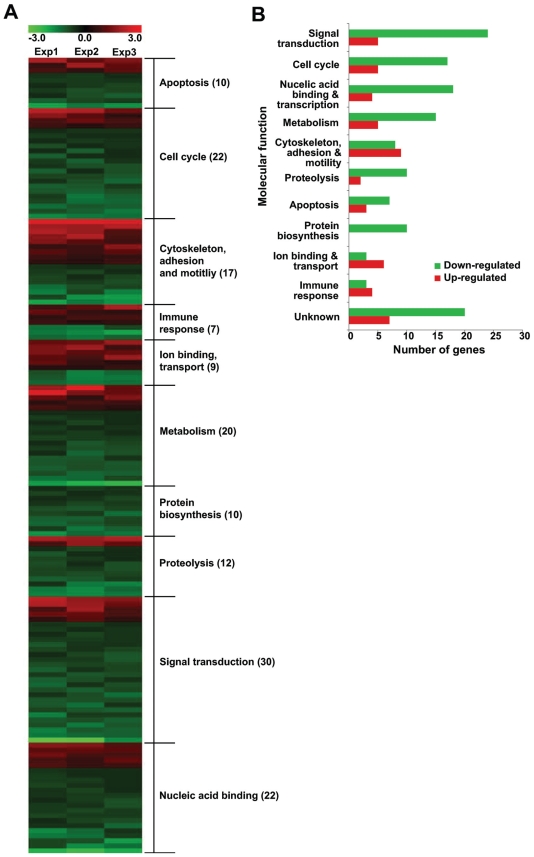
Functional categories of genes affected by TPPII knockdown **A**) Functional classification of the genes that are differentially regulated by TPPII knockdown. Heatmap showing modulated genes clustered into specific biological function based on Affymetrix annotation and GO classification. **B**) Graphical representation of the distribution of modulated genes in to functional groups.

In order to identify biological functions that are affected by the loss of TPPII, the 185 stringently modulated genes were classified into functional categories according to their Affymetrix and GO annotations (Supplementary information Table S1). Genes belonging to similar functional categories were then clustered to identify the affected biological functions (Fig. 3). This functional categorization revealed that the TPPII affected genes belong to families involved in the regulation of apoptosis, cell cycle, cytoskeleton, proteolysis, signal transduction, immune responses, ion transport and metabolism (Fig. 3). Twenty-seven out of 185 genes lack functional annotation.

To identify significantly affected biological functions, the GO database was searched using the set of differentially regulated genes. The GO Tree Machine available within WebGestalt was used to associate genes with GO terms. Enrichment of genes in each category was evaluated by Fisher’s exact test, which assigns a *P*-value indicating the statistical probability of the number of genes observed compared to the expected number, and categories with *P*-value <0.05 were identified as significantly affected. Table 1 shows a non-redundant list of significantly affected GO categories found under the Biological Process, Molecular Function or Cellular Component branches of the GO tree. Biological processes associated with cytoskeleton organization, cell cycle, cell death, immune responses and metabolism were significantly affected by TPPII knockdown. Under Molecular Function the largest number of regulated genes was found in categories associated with kinase activity, while the Cellular Components showed cytoplasmic proteins, particularly proteins involved in the microtubule and centrosome organization.

### KEGG pathway analysis

**Figure 4:**
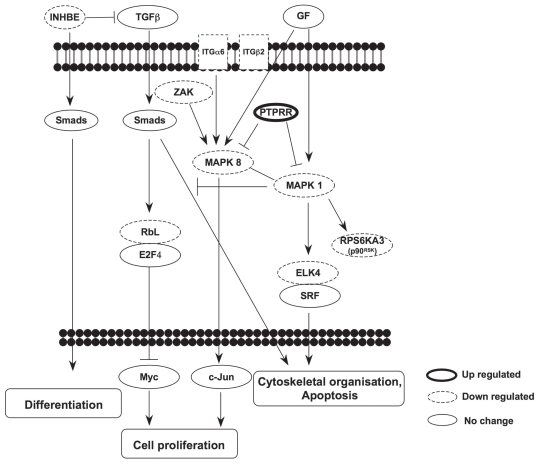
Schematic representation of the signaling pathways affected by TPPII knockdown KEGG pathway analysis of the differentially regulated genes demonstrates enrichment of components of the MAPK, Focal adhesion and TGF-β signaling. The annotated relationship between the regulated gene products is illustrated as activation (↓) or inhibition (⊥). Genes that are up- and down-regulated in TPPII knockdown cells are indicated by thick and dash-line boarders, respectively.

To identify the molecular events regulated by TPPII, affected genes were overlaid onto the KEGG pathway database. This analysis identified 63 KEGG pathways containing one or more stringently modulated genes. Statistically enriched pathways (P-value <0.05) are listed in Table 2. Three signal transduction pathways: Mitogen-activated protein kinase (MAPK) signaling (*P* = 0.0195), Focal adhesion (*P* = 0.0184) and TGF-β signaling (*P* = 0.028) and some amino acid metabolic pathways were found in this group. The MAPK signaling pathway is activated by a variety of extracellular stimuli and regulates a broad array of biological processes, including Focal adhesion and TGF-β signaling that were significantly affected by TPPII knockdown. Interestingly, many of the kinases of this pathway were down-regulated while the phosphatase, Protein tyrosine phosphatase, receptor type, R (PTPRR) was up-regulated suggesting that multiple events cooperate to inactivate the signaling cascade. A simplified scheme of the pathways identified by genes that are differentially expressed upon TPPII knockdown is shown in Figure 4.

### Protein interaction networks

The annotated molecular interactions of proteins encoded by the differentially regulated genes were used to interpret the observed transcriptional changes in a cellular context. For this analysis we took advantage of the human interactome data from BioGRID version 2.0.31 that compiles filtered protein-protein interactions from all currently available databases. Physical interactions are visualized as protein interaction maps, or networks, that consist of nodes (circles) symbolizing proteins, and edges (lines) representing biological relationship between two proteins.

**Figure 5:**
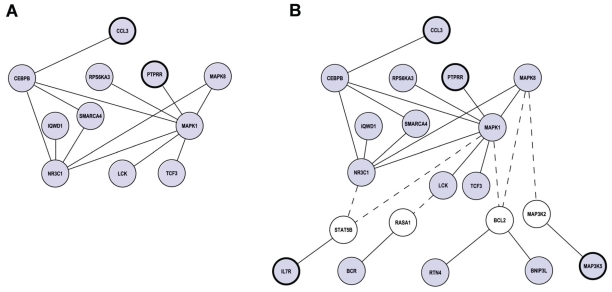
Members of the MAPK signaling cascade act as protein hubs of the interaction networks affected by TPPII knockdown **A**) Network derived from the binary interactions of the gene products that are differentially regulated by TPPII expression. Proteins are represented as nodes (circles) and interactions are represented as connecting edges (lines). Up-regulated and down-regulated genes are indicated as nodes with thick and thin borders respectively. **B**) Extended network including the gene products identified by the two-step search described in the Results section. Non-regulated intermediates are indicated by white circles and are connected to the original network by dashed lines.

Mapping of the 185 significantly affected genes onto the BioGRID dataset resulted in a small network of 11 genes including several components of the MAPK pathway that was also identified by the KEGG pathway analysis (Fig. 5A). To further recognize possible upstream and downstream components of the network, we performed a two-step search aiming to identify regulated genes that might have been disconnected due to no change of the connecting node. To this end, we first extracted all the interacting partners of the 11 hits mapped to the network and then listed all their interactions. From the complex network generated by this second level search we selected all the hits that were significantly regulated by TPPII knockdown. This resulted in the identification of 9 genes whose products are connected to the original network via non-regulated intermediates (Fig. 5B). The 20 interacting proteins identified by this analysis are all members of the MAPK signaling cascade, further confirming that MAPKs are central hubs in the molecular interactions regulated by TPPII.

## Discussion

Our earlier observation that overexpression of the cytosolic peptidase TPPII in epithelial and lymphoid cells correlates with abnormalities of centrosome and mitotic spindle and with genomic instability prompted us to undertake an in depth analysis of the role of this enzyme in the regulation of the cellular functions. Using a global approach based on the identification of genes affected by TPPII knockdown we have now found that TPPII is involved in a broad variety of cellular processes, including the cell cycle, apoptosis and cytoskeleton remodeling, all of which are directly or indirectly involved in the regulation of genomic stability. These findings are in line with a recent report demonstrating that TPPII knockout mice show multiple defects associated with inappropriate activation of death programs and cellular senescence (Huai et al. 2008). Our findings suggest that the pleiotropic effect of TPPII involves the regulation of MAPK signaling. Several MAP kinases were found to be down-regulated while the PTPRR phosphatase, an inhibitor of the pathways, was up-regulated in cells transduced with TPPII specific shRNAs, suggesting that different molecular events concur to silence this signaling pathways upon TPPII knockdown.

MAPK signaling is activated by Ras-family Guanosine triphosphatases (GTPases) and other protein kinases, and connects extracellular signals to transcription factors and other effectors that regulate many cellular programs such as cell proliferation, cell death and cytoskeleton remodeling. The activity of small GTPases is in turn regulated by Guanine exchange factors (GEFs) and GTPase activating proteins (GAPs) that play important roles in cell cycle and motility (Pruitt and Der, 2001). Two Ras GTPase regulators, RAS GTPase activating protein-1 (RASA1) and Breakpoint cluster region (BCR), which contains both Rho-GEF and Rho-GAP domains (Boguski and McCormick, 1993), were down-regulated by TPPII knockdown.

MAPKs are also activated by Src-like protein tyrosine kinases, such as Lymphocyte-specific protein tyrosine kinase (LCK) (Ettehadieh et al. 1992) that was found among the gene down-regulated by TPPII knockdown. Thus, several upstream regulators of MAP kinases were affected by TPPII silencing.

Two kinases, MAPK1 (Extracellular signal regulated kinase-2, ERK2) and MAPK8 (c-Jun N-terminal kinase, JNK) appear to act as central hubs in the interaction network regulated by TPPII. Several downstream effectors of these MAPKs, including the transcription factors signal transducer and activator of transcription 5B (STAT5B), Transcription factor 3 (TCF3) and CCAAT/enhancer binding protein (CEBPβ) were also down-regulated. Both TCF3 and STAT5B are targets of MAPK1 and were earlier shown to be involved in lymphocyte development and differentiation (Kee et al. 2000; Yao et al. 2006). TCF3 is a basic helix- loop-helix E2A transcription factor (Zhao et al. 2001). Though the function of CEBPβ is more complex, it is noteworthy that mammary epithelial cells lacking CEBPβ show decreased proliferation and increased rate of apoptosis (Robinson et al. 1998). NR3C1 (nuclear receptor subfamily 3, group C, member-1), a glucocorticoid receptor that appears to be involved in maintaining cell homeo-stasis (Revest et al. 2005) is another MAPK-regulated transcription factor that, under certain conditions, may also induce apoptosis (Tolosa et al. 1998). CEBPβ, STAT5 and NR3C1 were shown to associate with ATP-dependent chromatin remodeling SWI/SNF complexes (Xu et al. 2007). The transcript of one SWI/SNF complex associated protein, SMARCA4 (SWI/SNF related, matrix associated, actin dependent regulator of chromatin, subfamily A, member 4) was also found to be down-regulated by TPPII knockdown.

High expression of TPPII is associated with centrosome abnormalities and the formation of multipolar mitotic spindles in HEK293 and BL cells, while knockdown of TPPII in BL cells results in the accumulation of polynucleated cells (Stavropoulou et al. 2005). The possibility that TPPII may affect these phenotypes through regulation of centrosome functions is substantiated by our present finding that several known regulators of the centrosome cycle, most notably the centrosome-associated Polo like kinase-1 (Plk1), the pericentriolar material-1 (PCM1) and several cytoskeletal components such as dynein II light chain and kinesin II, were found amongst the genes consistently modulated upon TPPII knockdown. Furthermore, up-regulation of cytoskeleton components such as ACTL8, TTN and MYBPC1 may contribute to the morphological changes and increased motility induced by TPPII overexpression in HEK293 cells.

In conclusion, we have found that TPPII down-regulation is accompanied by coherent changes in the expression levels of genes whose products mediate critical cellular functions. Among the affected genes are several members of the MAPK signaling pathway that is a common regulator of the phenotypic effects induced by changes in TPPII expression. It remains to be seen how the activity of TPPII impacts on the regulation of transcription. Previous studies have shown that TPPII is involved in antigen processing and is the only cytosolic peptidase capable of processing proteasome products longer than 14 residues (Glas et al. 1998; Reits et al. 2004; York et al. 2006) but specific protein substrates have not been identified. Based on our findings it is tempting to speculate that those may include one or more pleiotropic transcription factors, with regulators of the MAPK cascade as primary candidates. Alternatively, TPPII may also target components of the cell cycle, apoptosis or cytoskeleton whose deregulation may in turn impact on MAPK signaling. The findings presented in this paper should be of value in guiding future work aiming to the identification of TPPII cellular substrates.

## Supplementary Material

Table S1 List of modulated genes on TPPII knockdown

## Figures and Tables

**Table 1:** Non-redundant GO categories enriched with differentially regulated genes.

GO category—Biological processes	Level	No. genes	P- value	Up-regulated	Down-regulated
**Cell organization and biogenesis**					
Maintenance of cell polarity	7	1	0.009		ANK1
Cytoskeleton organization	7	1	0.035	TTN	
Axon extension		1	0.036		RTN4
**Cell communication**					
Ras protein signal transduction	7	2	0.024	PLD1	LCK
Activation of MAPKK activity	9	1	0.036		ZAK
**Cell cycle**	4	11	0.030	HGF, TTN,	DNAJA2, USP16, ATF5, LCK, ZAK, PLK1, MAPK1, RBL2, SESN2
**Cell death**	6	5	0.014	IFNα2	LCK, ZAK, MAPK1, BNIP3L
**Cell metabolism**	4	59	0.047		
Pyrimidine nucleotide metabolism	7	2	0.023		DCK, CMPK
Establishment and maintenance of chromatin	8	5	0.027	HIST1H2AC	HMG20A, BCOR, TBL1XR1, SETD7,
**Response to stimulus**	2	20	0.015		
Response to stress	3	13	0.008	HGF, IFNα2, TTN, TLR10	CEBPβ, NR3C1, HIG2, ITGβ2, ZAK, MAPK1, MAPK8, CCL22, EDEM1
Chemotaxis	4	4	0.016	PLD1	ITGβ2, MAPK1, CCL22
**GO category—Molecular functions**					
**Protein binding**	3	49	0.018		
Protein kinase binding	6	3	0.016	PTPRR	ITGβ2, LCK,
Rab GTPase binding	8	2	0.009		RAB3GAP1, SYTL1
**Catalytic activity**					
Receptor signaling/MAPK activity	3	4	0.029		GNAZ, ZAK, MAPK1, MAPK8
Nucleotide kinase activity	6	4	0.000		DCK, AK2, MPP1, CMPK
**GO category—Cellular components**					
**Cytoplasmic proteins**	6	34	0.027		
Integrin complex	9	2	0.021		ITGα6, ITGβ2
Contractile fibers	8	2	0.046	MYBPC1, TTN	
Microtubule and cytoskeleton	6	6	0.017		NUDT21, PLK1, LCK, PCM1, KNS2, MAP1LC3B
Microtubule organizing center/centrosome	8	4	0.002		NUDT21, PLK1, LCK, PCM1
**Membrane proteins**					
Nuclear envelope	10	3	0.044		GNAZ, RTN4, BNIP3L
ER membrane	12	2	0.033		RTN4, EDEM1

**Table 2:** KEGG pathways that are significantly enriched with differentially regulated genes upon TPPII knockdown.

KEGG pathways	No. of genes		P-value	Up-regulated	Down-reguated
	Observed	Expected			
**Cell communication**					
Focal adhesion	5	1.49	0.018	HGF, TTN	ITGα6, MAPK1, MAPK8
**Signal transduction**					
Cytokine-cytokine receptor interaction	6	1.87	0.012	TNFRSF21, HGF, IL7R, IFNα2	CCL22, INHBE
MAPK signaling	6	2.08	0.020	PTPRR	ELK4, ZAK, MAPK1, MAPK8, RPS6KA3
TGF-beta signaling	3	0.64	0.028		MAPK1, RBL2, INHBE
**Metabolism**					
Citrate cycle	2	0.21	0.022		CS, IDH2
Alanine and aspartate metabolism	2	0.23	0.025		ASS1, PDHX
Vitamin B6 metabolism	1	0.03	0.042		PSAT1
