# Supplementary material for: The MAPK Signaling Cascade is a Central Hub in the Regulation of Cell Cycle, Apoptosis and Cytoskeleton Remodeling by Tripeptidyl-Peptidase II
Source: Gene Regul Syst Bio. 2008 Nov 24;2:253–65. doi: 10.4137/grsb.s882 (PMC2733081; doi:10.4137/grsb.s882)
Supplement: Table S1 — List of modulated genes on TPPII knockdown [file Table_S1.pdf]

Table 1. List of modulated genes on TPPII knockdown.

Probe set	Gene Symbol	Gene title	Avg-SLR	SD-SLR	Fold change
**Apoptosis (10)**					
211338_at	IFNA2	Interferon alpha 2	1.57	0.40	2.96
242714_at	MAP3K5	Mitogen-activated protein kinase kinase kinase 5	1.27	0.60	2.41
214581_x_at	TNFRSF21	Tumor necrosis factor receptor superfamily member 21	0.87	0.25	1.82
216321_s_at	NR3C1	Nuclear receptor subfamily 3 group C member 1	-0.60	0.10	1.52
211509_s_at	RTN4	Reticulon 4	-0.67	0.06	1.59
212501_at	CEBPB	CCAAT/enhancer binding protein (C/EBP) beta	-0.67	0.15	1.59
226297_at	HIPK3	Homeodomain interacting protein kinase 3	-0.80	0.10	1.74
202803_s_at	ITGB2	Integrin beta 2	-0.83	0.31	1.78
221478_at	BNIP3L	BCL2/adenovirus E1B 19kda interacting protein 3-like	-0.87	0.21	1.82
213883_s_at	TM2D1	Beta-amyloid binding protein precursor	-1.67	0.25	3.17
**Cell cycle (23)**					
226841_at	MPEG1	Macrophage expressed gene 1	1.87	0.49	3.65
215723_s_at	PLD1	Phospholipase D1 phophatidylcholine-specific	1.10	0.50	2.14
209960_at	HGF	Hepatocyte growth factor (hepapoietin A; scatter factor)	1.03	0.23	2.05
212096_s_at	MTUS1	Mitochondrial tumor suppressor 1	0.67	0.15	1.59
224621_at	MAPK1	Mitogen-activated protein kinase 1 (LOC440806)	-0.57	0.06	1.48
202174_s_at	PCM1	Pericentriolar material 1	-0.57	0.12	1.48
238075_at	CHEK1	Cell cycle checkpoint kinase	-0.60	0.10	1.52
225662_at	ZAK	Sterile alpha motif and leucine zipper containing kinase AZK	-0.67	0.21	1.59
223195_s_at	SESN2	Sestrin 2, Hypoxia induced gene 95	-0.70	0.35	1.62
226994_at	DNAJA2	Dnaj (Hsp40) homolog subfamily A member 2	-0.70	0.35	1.62
202240_at	PLK1	Polo-like kinase 1 (Drosophila)	-0.80	0.26	1.74
215260_s_at	TCF3	Transcription factor 3 (E2A immunoglobulin enhancer binding factors E12/E47)	-0.80	0.17	1.74
223238_s_at	PBRM1	Polybromo 1 (mitosis)	-0.83	0.31	1.78
202315_s_at	BCR	Breakpoint cluster region	-0.87	0.12	1.82
204891_s_at	LCK	Lymphocyte-specific protein tyrosine kinase	-0.90	0.30	1.87
218507_at	HIG2	Hypoxia-inducible protein 2	-0.93	0.35	1.91
226191_at	GSK3B	Glycogen synthase kinase 3 beta	-1.00	0.10	2.00
204999_s_at	ATF5	Activating transcription factor 5	-1.07	0.35	2.09
226048_at	MAPK8	Mitogen-activated protein kinase 8	-1.10	0.26	2.14
222633_at	TBL1XR1	Transducin (beta)-like 1X-linked receptor 1 (HDAC 3 sub)	-1.13	0.15	2.19
219812_at	STAG3	Stromal antigen 3	-1.13	0.25	2.19
225953_at	P15RS	Cyclin-dependent kinase inhibitor-related protein	-1.20	0.26	2.30
212331_at	RBL2	Retinoblastoma-like 2 (p130)	-1.43	0.15	2.70
**Cytokeleton, adhesion, motility (17)**					
214957_at	ACTL8	Actin like protein	2.57	0.06	5.92
214087_s_at	MYBPC1	Myosin binding protein C slow type	1.93	0.15	3.82
205337_at	DCT	Dopachrome tautomerase (dopachrome delta-isomerase tyrosine-related protein 2)	1.63	0.57	3.10
205114_s_at	CCL3	Chemokine (C-C motif) ligand 3	1.53	0.60	2.89
215565_at	DTNB	Dystrobrevin	1.13	0.35	2.19
219737_s_at	PCDH9	Protocadherin 9	1.03	0.49	2.05
205352_at	SERPINI1	Serine (or cysteine) proteinase inhibitor clade I (neuroserpin) member 1	1.00	0.17	2.00
217853_at	TNS3	Tensin-like SH2 domain containing 1	0.70	0.10	1.62
208195_at	TTN	Titin	0.63	0.15	1.55
212877_at	KLC1	Kinesin 2 60/70kda	-0.57	0.06	1.48

208786_s_at	MAP1LC3B	Microtubule-associated protein 1 light chain 3 beta	-0.67	0.15	1.59
239329_at	RAB21	RAB21 member RAS oncogene family	-0.70	0.17	1.62
201656_at	ITGA6	Integrin alpha 6	-0.80	0.10	1.74
228603_at	ACTR3	ARP3 actin-related protein 3 homolog (yeast)	-1.00	0.26	2.00
203763_at	DYNC2LI1	Dynein 2 light intermediate chain	-1.27	0.40	2.41
208353_x_at	ANK1	Ankyrin 1 erythrocytic	-1.43	0.55	2.70
213241_at	PLXNC1	Plexin C1	-1.67	0.25	3.17
**Immune response (6)**					
204747_at	IFIT3	Interferon-induced protein with tetratricopeptide repeats 3	0.83	0.32	1.78
223751_x_at	TLR10	Toll-like receptor 10	0.80	0.17	1.74
204439_at	IFI44L	Interferon-induced protein 44-like	0.63	0.15	1.55
221349_at	VPREB1	Pre-B lymphocyte gene 1	-1.17	0.32	2.24
207861_at	CCL22	Chemokine (C-C motif) ligand 22	-1.63	0.25	3.10
212314_at	KIAA0746	KIAA0746 protein	-1.23	0.15	2.35
**Metal ion binding & Ion Transport (9)**					
231935_at	ARPP-21	Cyclic AMP-regulated phosphoprotein 21 kd nucleic acid binding	1.43	0.32	2.70
242625_at	RSAD2	Radical S-adenosyl methionine domain containing 2	1.40	0.56	2.64
221646_s_at	ZDHHC1	Zinc finger DHHC domain containing 11	1.27	0.21	2.41
234034_at	KCNMB4	Potassium large conductance calcium-activated channel	1.27	0.47	2.41
219360_s_at	TRPM4	Transient receptor potential cation channel	0.80	0.36	1.74
213164_at	SLC5A3	Solute carrier family 5 (inositol transporters) member 3	0.57	0.12	1.48
212690_at	DDHD2	DDHD domain containing 2	-1.20	0.30	2.30
219270_at	CHAC1	Chac cation transport regulator-like 1	-1.30	0.26	2.46
202919_at	PREI3	Preimplantation protein 3	-1.30	0.10	2.46
**Meabolism (20)**					
209994_s_at	ABC1	ATP-binding cassette sub-family B (MDR/TAP) 1	1.90	0.75	3.73
207819_s_at	ABC4	ATP-binding cassette sub-family B (MDR/TAP) 4	1.77	0.74	3.40
1554474_a_at	MOXD1	Monooxygenase DBH-like 1	1.10	0.46	2.14
211138_s_at	KMO	Kynurenine 3-monooxygenase (kynurenine 3-hydroxylase)	0.77	0.15	1.70
223220_s_at	PARP9	Poly (ADP-ribose) polymerase family member 9	0.70	0.20	1.62
210046_s_at	IDH2	Isocitrate dehydrogenase 2 (NADP+) mitochondrial	-0.50	0.00	1.41
203302_at	DCK	Deoxycytidine kinase	-0.60	0.10	1.52
208660_at	CS	Citrate synthase	-0.60	0.17	1.52
212174_at	AK2	Adenylate kinase 2	-0.60	0.10	1.52
225007_at	G3BP	Ras-GTPase-activating protein SH3-domain-binding protein	-0.60	0.10	1.52
201660_at	ACSL3	Acyl-coA synthetase long-chain family member 3	-0.73	0.06	1.66
223062_s_at	PSAT1	Phosphoserine aminotransferase 1	-0.77	0.25	1.70
202119_s_at	CPNE3	Copine III	-0.83	0.12	1.78
209610_s_at	SLC1A4	Glutamate/neutral amino acid transporter	-0.87	0.35	1.82
217989_at	HSD17B11	hydroxysteroid (17-beta) dehydrogenase 11	-0.87	0.23	1.82
203067_at	PDHX	Pyruvate dehydrogenase complex component X	-0.97	0.06	1.95
217870_s_at	CMPK	UMP-CMP kinase	-1.00	0.10	2.00
201313_at	ENO2	Enolase 2 (gamma neuronal)	-1.07	0.15	2.09
226894_at	SLC35A3	(UDP-N-acetylglucosamine (UDP-glcnac) transporter)	-1.10	0.26	2.14
207076_s_at	ASS1	Argininosuccinate synthetase	-2.07	0.25	4.19
**Protein biosynthesis & transport (11)**					
200699_at	KDELR2	KDEL (Lys-Asp-Glu-Leu) ER protein retention receptor 2	-0.60	0.10	1.52
227808_at	DNAJC15	Dnaj (Hsp40) homolog subfamily D member 1	-0.63	0.15	1.55
204169_at	IMPDH1	IMP (inosine monophosphate) dehydrogenase 1	-0.63	0.15	1.55

226119_at	PCMTD1	Protein-L-isoaspartate (D-aspartate) O-methyltransferase domain containing 1	-0.67	0.15	1.59
231894_at	SARS	Seryl-trna synthetase	-0.70	0.10	1.62
213310_at	EIF2C2	Eukaryotic translation initiation factor 2C 2	-0.90	0.10	1.87
227134_at	SYTL1	Synaptotagmin-like 1	-0.93	0.32	1.91
212812_at	SERINC5	Serine incorporator 5	-1.00	0.17	2.00
222627_at	VPS54	Vacuolar protein sorting 54 (yeast)	-1.00	0.20	2.00
209921_at	SLC7A11	(Cationic amino acid transporter y+ system) member 11	-1.07	0.35	2.09
209628_at	NXT2	Nuclear transport factor 2-like export factor 2	-1.43	0.25	2.70
**Proteolysis (12)**					
1553499_s_at	SERPINA9	Serine (or cysteine) proteinase inhibitor clade A (alpha-1 antiproteinase antitrypsin)	1.97	0.15	3.91
228055_at	NAPSB	Napsin B aspartic peptidase pseudogene	1.27	0.40	2.41
203279_at	EDEM1	ER degradation enhancer mannosidase alpha-like 1	-0.60	0.17	1.52
226106_at	RNF141	Ring finger protein 141	-0.67	0.29	1.59
226921_at	UBR1	Ubiquitin protein ligase E3 component n-recognin 1	-0.77	0.23	1.70
223132_s_at	TRIM8	Tripartite motif-containing 8	-0.80	0.10	1.74
220419_s_at	USP25	Ubiquitin specific protease 25	-0.83	0.06	1.78
228822_s_at	USP16	Ubiquitin specific protease 16	-0.93	0.21	1.91
226343_at	DPP8	Dipeptidyl peptidase 8 isoform 3, Similar to NP_932064.1	-0.93	0.21	1.91
226140_s_at	OTUD1	OTU domain containing 1	-1.17	0.49	2.24
226326_at	PCGF5	Polycomb group ring finger 5	-1.40	0.20	2.64
203374_s_at	TPP2	Tripeptidyl peptidase II	-1.50	0.10	2.83
**Signal transduction (28)**					
206084_at	PTPRR	Protein tyrosine phosphatase receptor type R	1.83	0.32	3.56
223343_at	MS4A7	Membrane-spanning 4-domains subfamily A member 7	1.73	0.40	3.32
205798_at	IL7R	Interleukin 7 receptor	1.30	0.62	2.46
238623_at	MAP3K4	Mitogen-activated protein kinase kinase kinase 4	1.23	0.58	2.35
209795_at	CD69	CD69 antigen (p60 early T-cell activation antigen)	1.13	0.21	2.19
201760_s_at	WSB2	WD repeat and SOCS box-containing 2	0.77	0.29	1.70
225572_at	FAM119A	CAMP responsive element binding protein 1	-0.60	0.10	1.52
209307_at	SWAP70	Switch-associated protein 70	-0.60	0.10	1.52
202442_at	AP3S1	Adaptor-related protein complex 3 sigma 1 subunit	-0.63	0.15	1.55
224596_at	SLC44A1	CDW92 antigen	-0.67	0.06	1.59
220351_at	CCRL1	Chemokine (C-C motif) receptor-like 1	-0.73	0.23	1.66
226370_at	KLHL15	Kelch-like 15 (Drosophila)	-0.73	0.12	1.66
213531_s_at	RAB3GAP1	RAB3 GTPase-activating protein	-0.77	0.25	1.70
204993_at	GNAZ	Guanine nucleotide binding protein (G protein) alpha Z polypeptide	-0.80	0.17	1.74
208781_x_at	SNX3	Sorting nexin 3	-0.83	0.06	1.78
217908_s_at	IQWD1	IQ motif and WD repeats 1	-0.83	0.25	1.78
226335_at	RPS6KA3	Ribosomal protein S6 kinase 90kda polypeptide 3	-0.87	0.15	1.82
226990_at	GPIAP1	GPI-anchored membrane protein 1	-0.87	0.25	1.82
210970_s_at	IBTK	Inhibitor of Bruton agammaglobulinemia tyrosine kinase	-0.90	0.30	1.87
202974_at	MPP1	Membrane protein palmitoylated 1	-0.93	0.15	1.91
225232_at	MTMR12	Phosphatidylinositol-3-phosphate associated protein	-0.93	0.06	1.91
227945_at	TBC1D1	TBC1(tre-2/USP6 BUB2 cdc16) domain family member 1	-0.97	0.35	1.95
218493_at	C16ORF33	Chromosome 16 open reading frame 33	-0.97	0.15	1.95
203884_s_at	RAB11FIP2	RAB11 family interacting protein 2 (class I)	-0.97	0.46	1.95
213379_at	COQ2	Coenzyme Q2 homolog, prenyltransferase (yeast)	-1.00	0.10	2.00
228662_at	SOCS7	Suppressor of cytokine signaling 7	-1.10	0.26	2.14
201691_s_at	TPD52	Tumor protein D52	-1.23	0.15	2.35
210587_at	INHBE	Inhibin beta E	-2.67	0.97	6.35

**Nucleic acid Binding (Replication, Transcription etc) (22)**					
209806_at	HIST1H2BK	Histone 1 h2bk	1.37	0.32	2.58
238447_at	RBMS3	RNA binding motif single stranded interacting protein	1.13	0.06	2.19
215071_s_at	HIST1H2AC	Histone 1 h2ac	1.03	0.23	2.05
224828_at	CPEB4	Cytoplasmic polyadenylation element binding protein 4	0.93	0.25	1.91
230795_at	HIST2H4	HIST2H4 histone 2 H4; complete cds	0.83	0.15	1.78
206102_at	GINS1	GINS complex subunit 1 (Psf1 homolog)	-0.53	0.06	1.45
218152_at	HMG20A	High-mobility group 20A	-0.57	0.12	1.48
224830_at	NUDT21	Nudix (nucleoside diphosphate linked moiety X)-motif 21	-0.63	0.12	1.55
212612_at	RCOR1	REST corepressor 1	-0.63	0.15	1.55
219126_at	PHF10	PHD finger protein 10	-0.63	0.15	1.55
219433_at	BCOR	BCL6 co-repressor	-0.67	0.06	1.59
208984_x_at	RBM10	RNA binding motif protein 10	-0.70	0.20	1.62
213720_s_at	SMARCA4	SWI/SNF related matrix associated actin dependent regulator of chromatin	-0.73	0.15	1.66
222476_at	CNOT6	CCR4-NOT transcription complex subunit 6	-0.73	0.06	1.66
225159_s_at	ELK4	ETS-domain protein (SRF accessory protein 1)	-0.73	0.06	1.66
224928_at	SETD7	SET domain containing (lysine methyltransferase) 7	-0.73	0.21	1.66
230194_at	LRPPRC	Leucine-rich PPR-motif containing	-0.80	0.35	1.74
212124_at	RAI17	Retinoic acid induced 17	-1.13	0.50	2.19
203640_at	MBNL2	Muscleblind-like 2 (Drosophila)	-1.13	0.38	2.19
206115_at	EGR3	Early growth response 3	-1.17	0.55	2.24
203361_s_at	MYCBP	C-myc binding protein	-1.23	0.40	2.35
1555801_s_at	ZNF533	Zinc finger protein 533	-2.17	0.21	4.49
**Unknown (27)**					
231223_at	CSMD1	CUB and Sushi multiple domains 1	1.83	0.50	3.56
221973_at	LOC150759	Hypothetical protein LOC150759	1.13	0.38	2.19
238429_at	TMEM71	Hypothetical protein FLJ33069	1.03	0.06	2.05
230451_at	C1ORF136	Chromosome 1 open reading frame 136	1.00	0.30	2.00
227966_s_at	CCDC74A /// CCDC74B	coiled-coil domain containing 74A /// 74B	0.90	0.10	1.87
228195_at	MGC13057	Hypothetical protein MGC13057	0.90	0.36	1.87
238674_at	---	---	0.57	0.12	1.48
212150_at	KIAA0143	KIAA0143 protein	-0.53	0.06	1.45
219563_at	C14ORF139	Chromosome 14 open reading frame 139	-0.67	0.12	1.59
224759_s_at	C12ORF23	Hypothetical protein MGC17943	-0.67	0.15	1.59
226276_at	TMEM167	transmembrane protein 167	-0.67	0.15	1.59
226810_at	C6ORF155	Chromosome 6 open reading frame 155	-0.67	0.12	1.59
208809_s_at	C6ORF62	Chromosome 6 open reading frame 62	-0.70	0.20	1.62
218187_s_at	C8ORF33	Hypothetical protein FLJ20989	-0.70	0.26	1.62
212731_at	ANKRD46	Ankyrin repeat domain 46	-0.80	0.26	1.74
212792_at	DPY19L1	Dpy-19-like 1	-0.80	0.10	1.74
222391_at	TMEM30A	Transmembrane protein 30A	-0.80	0.17	1.74
225224_at	C20ORF112	---	-0.83	0.12	1.78
224844_at	SLAIN2	SLAIN motif family, member 2	-0.87	0.21	1.82
228812_at	---	Transcribed locus weakly similar to NP_689672.2 MGC45438	-0.87	0.21	1.82
217906_at	KLHDC2	Kelch domain containing 2	-0.90	0.26	1.87
227638_at	KIAA1632	KIAA1632	-0.93	0.31	1.91
231035_s_at	---	---	-0.93	0.12	1.91
1562610_at	IMAGE:483	Homo sapiens clone IMAGE:4830466	-1.00	0.36	2.00
221477_s_at	MGC5618	FLJ43505 protein	-1.13	0.12	2.19
228242_at	---	---	-1.23	0.42	2.35
224502_s_at	KIAA1191	KIAA1191 protein	-1.53	0.06	2.89
